# Uptake and Persistence of Safer Conception Strategies Among South African Women Planning for Pregnancy

**DOI:** 10.1007/s10461-024-04475-z

**Published:** 2024-09-06

**Authors:** Oluwaseyi O. Isehunwa, Manjeetha Jaggernath, Yolandie Kriel, Christina Psaros, Mxolisi Mathenjwa, Kathleen E. Hurwitz, Kara Bennett, Patricia M. Smith, David R. Bangsberg, Jeanne M. Marrazzo, Jessica E. Haberer, Jennifer A. Smit, Lynn T. Matthews

**Affiliations:** 1 Division of Infectious Diseases, Heersink School of Medicine, University of Alabama at Birmingham, 703 19Th Street South, Birmingham, AL 35233 USA; 2 Wits MRU (MatCH Research Unit), Department of Obstetrics and Gynaecology, Faculty of Health Sciences, University of the Witwatersrand, Durban, South Africa; 3 Department of Psychiatry, Massachusetts General Hospital, Boston, USA; 4 Department of Medicine, Harvard Medical School, Boston, USA; 5 Epidemiology and Prevention Department, Centre for the AIDS Programme of South Africa (CAPRISA), Durban, South Africa; 6 Target RWE, Durham, NC USA; 7 Vin University College of Health Sciences, Hanoi, Vietnam; 8 Center for Global Health, Massachusetts General Hospital, Boston, USA

**Keywords:** Safer conception strategies, Persistence, Women planning for pregnancy, HIV prevention, South Africa

## Abstract

Safer conception strategies can minimize HIV acquisition during periconception periods among women living in HIV-endemic areas. We examined uptake and predictors of persistent use of the same safer conception strategy among a cohort of HIV-uninfected South African women ages 18–35 years planning for pregnancy with a partner living with HIV or of unknown HIV-serostatus. The safer conception strategies we evaluated included oral PrEP, condomless sex limited to peak fertility, and waiting for a better time to have a child (until, for example, the risks of HIV acquisition are reduced and/or the individual is prepared to care for a child); persistence was defined as using the same safer conception strategy from the first visit through 9 months follow-up. Modified Poisson regression models were used to examine predictors of persistent use of the same strategy. The average age of 227 women in our cohort was 24.6 (range: 18.0, 35.7) years. In this cohort, 121 (74.2%) women reported persisting in the same strategy through 9 months. Employment and HIV knowledge were associated with the persistent use of any strategy. Our results highlight the need to provide safer conception services to women exposed to HIV during periconception periods. Findings also offer some insights into factors that might influence persistent use. Further research is needed to better understand how to involve male partners and how their involvement might influence women’s consistent use of safer conception strategies during periconception periods.

## Introduction

In many HIV-endemic sub-Saharan countries like South Africa, heterosexual contact is an important driver of HIV transmission [1, 2]. Many women choosing to conceive children have a partner with HIV thus increasing risks of HIV acquisition and transmission to their infants [3, 4]. Additionally, women in HIV-endemic areas may conceive with an unknown serostatus partner, which may also expose them to HIV. To reduce periconception HIV risks, several “safer conception” strategies prevent HIV while allowing for conception, including suppressive antiretroviral therapy (ART) for partners with HIV, limiting condomless sex to peak fertility, sperm washing with artificial insemination, and pre-exposure prophylaxis (PrEP) for uninfected people [5–7]. Safer conception care has not been integrated into the standard of care [8, 9], and many women remain vulnerable to acquiring HIV during periconception periods; perinatal transmission of HIV infection among children is still high in sub-Saharan Africa [10–12].

Previous observational studies and demonstration projects from sub-Saharan African countries suggest inconsistent uptake when safer conception strategies are offered. In a large demonstration project involving heterosexual HIV-serodifferent couples from Kenya and Uganda, among those with fertility desire and plans for pregnancy, participants had access to oral PrEP, ART, and timed condomless sex, while referral information for sperm washing and other safer conception options were provided [13]. Among women with immediate fertility desires, 37% reported using oral PrEP, 14% ART, and 30% timed condomless sex at their one-year follow-up visit [13]. Similarly, in a prospective cohort study of 400 people with HIV with the majority having fertility desires or plans for pregnancy, 31% of those whose partner’s HIV status was negative or unknown serostatus reported the use of timed condomless sex at any time during the study period [14]. None reported the use of sperm washing among those whose partner was HIV-negative or unknown [14]. More recent studies on oral PrEP use among women planning for pregnancy with a partner with HIV or of unknown serostatus, or ART use by a partner with HIV have ranged from low to high uptake, and consistent use remains a challenge [3, 9].

The uptake of safer conception strategies could be influenced by several factors including knowledge and awareness of safer conception strategies, income, the number of living children, childbearing stigma, social support, provider training and communication about safer conception, relationship status with a partner (e.g., casual or primary partner), partner’s HIV status/staging of HIV, the degree of control over sexual decision making in the relationship, and partner being in agreement with safer conception [13, 15–18]. Persistence of safer conception strategy use is crucial to minimize the risk of HIV transmission during periconception and pregnancy periods and may inform counseling approaches in stretched healthcare systems. More importantly, promoting high persistence on safer conception strategies during periods of HIV risk—prevention-effective persistence, may support the prevention of HIV acquisition and perinatal transmission [19]. However, studies on the uptake and persistent use of safer conception strategies among women during pregnancy or while planning for pregnancy in HIV-endemic settings in sub-Saharan Africa are limited.

Using data from a prospective cohort study, we assess uptake and predictors of persistent use of safer conception strategies among women planning for pregnancy with a partner with HIV or of unknown HIV-serostatus.

## Methods

### Study Design and Study Population

Data for this analysis comes from the ZINK, or *Zivikele ngaphambi kokukhulelwa*—meaning *“Protecting Yourself Before Pregnancy*”–study. This single-arm longitudinal safer conception intervention study comprised 330 HIV-uninfected women from KwaZulu-Natal, South Africa planning for pregnancy with a partner with HIV or of unknown serostatus. The primary aim of the ZINK study was to assess oral PrEP uptake and adherence during periconception and pregnancy [20]. Women were eligible to participate if they were 1) aged 18–35 years, 2) were likely to be fertile based on responses to reproductive history assessment, 3) not on a long-acting family planning method, 4) reported personal or partner’s plans for a child within the next 12 months with a 5) partner with HIV or serostatus unknown, 6) fluent in English or isiZulu, and 7) able to provide informed consent [20]. Women were excluded from the study if they were 1) living with HIV, 2) pregnant, 3) hospitalized within 30 days before entry into the study, or receiving treatment for an active illness that could interfere with study participation [20]. Women were enrolled from November 2017 to January 2020 and followed quarterly for a minimum of 12 months. Women who became pregnant during the study were followed through to pregnancy outcomes for a maximum of 24 months. In addition to oral PrEP, women were counseled on safer conception strategies including partner testing and serostatus HIV disclosure, limiting condomless sex to peak fertility, delaying sex without condomless sex to peak fertility, delaying sex without condoms until partner achieves HIV RNA suppression or on antiretroviral therapy (ART) for 6 months for partners with HIV, and waiting to have a child. We also offered couples-based HIV counseling and testing as a safer conception strategy on site, however no woman brought a partner to the site for this service.

### Data Collection

Women completed an enrollment questionnaire to assess socio-demographics (including age, education attainment, employment status, marital status, social support [21], and income), reproductive history (including the number of prior pregnancies in a lifetime, any child with the main partner, number of sexual partners, condom use during last sex with the main partner, HIV status of main partner), lifestyle and health-related history (including alcohol consumption, and depression) and other constructs based on a conceptual framework for periconception risk behavior (including HIV knowledge [22], HIV risk perception [23], HIV stigma [24], dyadic trust [25, 26], and sexual relationship power [27]. Safer conception behaviors were assessed at each quarterly visit (every 3 months) through a brief safer conception card which women were asked to complete to indicate their safer conception strategy selections. The safer conception counseling was first offered at the second visit (within 1 month of enrolment), and at each study visit thereafter for those who remained non-pregnant. Women who became pregnant received counseling around HIV risk reduction in the context of pregnancy [20].

## Measures

### Use and Persistent Use of Safer Conception Strategies

Two main outcomes were assessed 1) use of safer conception strategies, which was defined as the proportion of women who self-reported using at least one safer conception strategy during the follow-up period -1, 3, 6, and 9 months, and 2) Self-reported persistent use of safer conception strategy, which was defined as the proportion of women who reported using the same safer conception strategy from 3 months through 9 months follow-up visit.

Women who were not pregnant were asked about the safer conception strategy used at each study visit by research staff. Their strategies were documented on a safer conception card: “Which safer conception strategies is the participant using?” with the following options available: “*Wait to have a child” (waiting for a better time to have a child until the risk of HIV acquisition and perinatal transmission are reduced*)*; “Disclosure of HIV status”; “Timed sex without condoms to peak fertility”; “Anti-Retroviral Therapy”; “oral Pre-exposure prophylaxis (oral PrEP)”; “Sperm washing”; “Other”; or “None*”. The use of a safer conception strategy was assessed from among the 6 safer conception strategies listed above. The strategies assessed for persistence were those that could be utilized longitudinally including waiting to have a child, timed sex without condoms to peak fertility, or oral PrEP (self-report) from the second visit. Persistence in ART for partners with HIV and sperm washing was not assessed due to the few numbers of people selecting those strategies. Likewise, persistence in HIV disclosure status by partner was not assessed as it is only expected to be a single event. Persistence was assessed among women who indicated they were using any of the three above safer conception strategies at the second visit (within 1 month of enrolment). Women were censored at pregnancy or loss to follow up.

### Sexual Relationship Power

Was assessed by the sexual relationship power scale (SRPS) [27] which includes 23 items that are scored as 2 subscales–relationship (15-item questions) and decision-making dominance (8-item questions). For example, women were asked the following questions about their relationship “*If I asked my partner to use a condom, he would get violent”, “Most of the time, we do what my partner wants to do”, “My partner might be having sex with someone else*” with 4-choice Likert scale responses. Example questions assessing decision-making dominance included “*Who usually has more say about whether you use condoms?” “In general who do you think has more power in your relationship?”, and “Who usually has more say about what types of sexual acts you do?”* Response options included “*Your partner”, “Both of you equally”, and “You”.* A composite score was derived. Possible scores ranged from 1 to 4, with higher scores indicating more power in the relationship in the area of relationship control and decision-making dominance.

### Dyadic Trust

Was assessed by the Dyadic Trust Scale [25], consisting of 8-item questions with 5-point Likert scale response options including *“My partner is primarily interested in his welfare”, “My partner is perfectly honest and truthful with me”, and “I feel that I can trust my partner completely”, “There are times when my partner cannot be trusted”, and “ I feel that I can trust my partner completely”.* Responses to each item were summed to give an overall dyadic trust score. Possible scores ranged from 13 to 40, with higher scores indicating higher levels of dyadic trust.

### HIV Knowledge

Was assessed with 24-item questions–15-item questions from the brief HIV questionnaire originally developed by Carey and Schroder [22], and 9-item questions developed by Matthews et al. [15]. For example, women were asked the following questions: “ *A person can get HIV by sharing a glass of water with someone who has HIV”, “ A person can get HIV by sitting in a hot tub or swimming pool with a person who has HIV”, “If a woman has HIV then her baby will always be born with HIV”, “If an HIV-positive man has sex without condoms with an HIV-negative woman even once, she will always catch the virus”*. Response options included *“Yes”, “No”, or “Don’t Know”.* An overall HIV score was derived by summing scores of the correct answers. Possible scores ranged from 0 to 24, with higher scores indicating higher knowledge about HIV spread, prevention, and impacts of HIV infection.

### HIV Stigma

Attributed to others was assessed with 12 item questions from the HIV Stigma Scale developed by Visser et al. [24]. For example, women were asked to respond to the following questions with options *“I agree” or “I disagree”: “Most people think getting HIV is a punishment for bad behavior”, “Most people think that having HIV is just a matter of bad luck”, “Most people would reject the friendship of someone with HIV”, and “Most people are afraid to be around people with HIV”.* Possible scores ranged from 0 to 12, with higher scores indicating a higher level of stigmatizing attitudes attributed to others.

### Perceived HIV Risk

Was assessed with 7 item questions from the Perceived HIV Risk scale originally developed by Napper et al. [23]including “*What is your gut feeling about how likely you are to get infected with HIV?”, “I worry about getting infected with HIV”, “I am sure I will not get infected with HIV”, and “I feel I am unlikely to get infected with HIV”.* Items were rated on Likert-type scales. Responses to each item were summed, and an overall score was computed. Possible scores ranged from 7 to 34 with higher scores indicating higher perceived HIV risk.

### Ethics

Ethics approval was obtained from the Human Research Ethics Committee at the University of the Witwatersrand (Johannesburg, South Africa), and the Institutional Review Board of Partners Healthcare (Boston, Massachusetts, USA). Women were reimbursed 250 ZAR (~ 20 US$) per study visit [20].

### Statistical Analysis

Descriptive statistics were used to summarize the baseline characteristics of study participants. Univariable and multivariable modified Poisson regression models were conducted to assess predictors of persistence on any form of safer conception strategy from 3 months through 9 months. The univariable analysis included the following variables: age (continuous variable), education (Grade 7- Grade 11, Grade 12, and post-grade 12), employment status (employed, not employed), income (< $116, $116-$232), prior pregnancies (0, 1, 2 +), having a child with main partner (yes, no), number of sexual partners past 3 months (0 or 1, 2 +), condom use at last sex with a main partner (yes, no), alcohol consumption (any, never), depression (≥ 1.75, < 1.75), and sexual relationship power, dyadic trust, social support, HIV knowledge, HIV stigma, and perceived HIV risk each treated as a continuous variable. Variables were selected for inclusion in the multivariable analysis based on a p-value of 0.25. The risk ratio with its 95% confidence interval was computed. A p-value of less than 0.05 was considered statistically significant. All analyses were performed using SAS version 9.4.

## Results

### Baseline Characteristics

Table 1 presents the baseline characteristics of eligible study participants. The mean age of women at baseline was 24.6 years. Slightly over a third had completed at least some form of tertiary education–university, college, or vocation (n = 105, 38.6%), while less than a third were employed (n = 71, 26.1%). Nearly half of the women had no pregnancy in their lifetime (n = 124, 45.6%) and twenty-one percent had had a child with their main partner. Most women were in a long-term relationship (for at least 6 months) with their partner (n = 250, 92.3%) and did not know the HIV status of their partner (n = 262, 96.7%).

**Table 1:** Baseline characteristics of eligible non-pregnant women (N = 272)

	Overall N = 272
**Demographics**	
Age, years (n = 272)	
Mean (SD)	24.6 (3.7)
Median (Min, Max)	24.2 (18.0, 35.7)
Education (n = 272)	
Grade 7 (Standard 5)—Grade 11 (Standard 9)	39 (14.3%)
Grade 12 (Standard 10)	128 (47.1%)
Post-grade 12 (University, College, Vocational)	105 (38.6%)
Currently employed (n = 272)	71 (26.1%)
Income, monthly (n = 200)	
< $116	61 (30.5%)
$116—$232	70 (35.0%)
> $232	69 (34.5%)
**Reproductive History**	
Prior pregnancies (n = 272)	
0	124 (45.6%)
1	103 (37.9%)
2 +	45 (16.5%)
Have had a child with main pregnancy partner (n = 271)	57 (21.0%)
**Relationship/Partner Characteristics**	
Sexual partners, past 3 months (n = 271)	
0 or 1	238 (87.8%)
2 +	33 (12.2%)
Relationship status with main pregnancy partner (n = 271)	
Ongoing casual/one-time encounter	3 (1.1%)
Boyfriend/main partner for < 6 months	8 (3.0%)
Boyfriend/main partner for ≥ 6 months	250 (92.3%)
Spouse or living as married ≥ 6 months	10 (3.7%)
Used condom at last sex (main pregnancy partner) (n = 271)	77 (28.4%)
HIV status of main pregnancy partner (n = 271)	
HIV negative	1 (0.4%)
HIV positive	8 (3.0%)
HIV serostatus unknown	262 (96.7%)
Sexual Relationship Power Scale ^a^ (n = 235)	
Mean (SD)	2.6 (0.4)
Median (Min, Max)	2.6 (1.0,3.5)
Dyadic Trust Score ^b^ (n = 270)	
Mean (SD)	29.4 (6.3)
Median (Min, Max)	29.0 (13.0, 40.0)
Social support ^c^ (n = 271)	
Mean (SD)	3.9 (0.2)
Median (Min, Max)	4.0 (2.3, 4.0)
**Health behavior/Health Characteristics**	
Any alcohol consumption, past year (n = 269)	140 (52.0%)
Depression ≥ 1.75 ^d^ (n = 268)	18 (6.7%)
**HIV knowledge, stigma, and risk**	
HIV Knowledge Score ^e^ (n = 270)	
Mean (SD)	15.5 (3.2)
Median (IQR)	16 (13.0, 18.0)
HIV Stigma Score ^f^ (n = 259)	
Mean (SD)	3.3 (3.6)
Median (IQR)	2 (0, 6.0)
Perceived HIV Risk ^g^ (n = 259)	
Mean (SD)	19.9 (3.1)
Median (IQR)	20 (18.0, 22.0)

SD, IQR, Min, Max represents standard deviation, interquartile range, minimum value and maximum value respectively

^a^Developed by Pulerwitz et al., Sexual Relationship Power score assesses the relationship power dynamics around relationship control and decision-making. Higher scores denoted a higher level of power in the relationship around relationship control and decision-making

^b^Dyadic Trust Scale was used to assess the level of trust a person has in their relationship. Higher scores denoted a higher level of trust in the relationship

^c^Duke-UNC Functional support scale was used to measure social support. Higher scores denoted higher levels of social support

^d^Hopkins Symptoms Checklist was used to assess depression. A cut off point of ≥ 1.75 was categorized as presence of depressive symptoms

^e^Adapted from Carey and Schroder, and Matthews et al. HIV knowledge score was used to assess a person’s knowledge about HIV spread, transmission, and prevention. Higher scores denoted higher levels of knowledge about HIV spread, transmission, and prevention

^f^Developed by Visser et al., HIV Stigma Scale was used to assess perceptions of HIV stigmatizing attitudes within the community. Higher scores denoted higher levels of HIV stigmatizing attitudes within the community

^g^Developed by Napper et al. Perceived HIV Risk scale was used to assess perceptions of their risk of acquiring HIV. Higher scores denoted higher levels of perception of risk of acquiring HIV

### Use of Safer Conception Methods at Different Visits

**Fig. 1:**
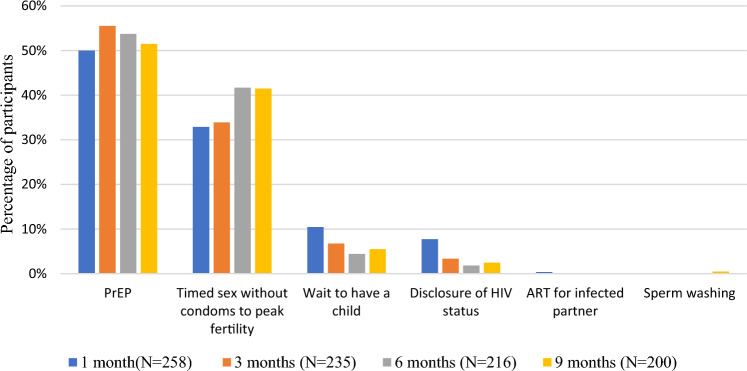
Proportion of HIV-uninfected women with an infected or unknown serostatus partner and personal or partner plans for pregnancy women reporting the use of safer conception methods at any study follow-up visit

Figure 1 shows the different safer conception methods and the proportion of women who reported planning to use the methods immediately after the first safer conception counseling (second visit), at 3 months, 6 months, and 9 months. At the second visit (within 1 month of enrolment), the number and proportion e of women who reported selecting the strategies of oral PrEP, timed sex without a condom to peak fertility, waiting to have a child, disclosure of HIV status, ART for an infected partner, or sperm washing were 129 (50%), 86 (33%), 27 (10%), 20 (8%), 1 (0.4%), and 0 (0%), respectively. At 3, 6, and 9 months, the number and proportion of women who reported using oral PrEP was: 130 (55%), 116 (54%), and 103 (52%); timed sex without condom to peak fertility – 80 (34%), 90 (42%), and 83 (42%); waiting to have a child – 16 (7%), 10 (4%), and 11 (6%); disclosure of HIV status – 8 (3%), 4 (2%), and 5 (3%) respectively. No woman reported ART for a partner with HIV as a safer conception strategy at the 3-, 6-, or 9-month follow-up visit, while only one woman reported sperm washing as a safer conception strategy utilized at the 9-month follow-up visit.

### Persistence Among Selected Safer Conception Strategies

Table 2 shows the proportion of women who persisted in the use of selected safer conception strategies from initiation at the second visit through 3 months, 6 months, and 9 months of follow-up: oral PrEP, timed sex without a condom to peak fertility, and waiting to have a child. The proportion of women who reported persisting with oral PrEP was 97% (109 of 112 women), 92% (85 of 92 women), and 86% (70 of 81 women) through 3,6, and 9 months, respectively. The proportion of women who reported persisting with timed sex without a condom to peak fertility was 84% (63 of 75 women), 76% (54 of 71 women), and 76% (48 of 63 women) through 3, 6, and 9 months, respectively. The proportion of women who reported persisting with waiting to have a child was 30%, 12%, and 12% through 3, 6, and 9 months respectively.

**Table 2:** Among women who initiated a safer conception strategy after the first safer conception counseling session, this table shows persistence in their use of that strategy from initiation at second visit through 3 months, 6 months, and 9 months of follow-up

	# of women who initiated safer conception strategy after first counseling	Persistence through 3 months n (%)	Persistence through 6 months n (%)	Persistence through 9 months n (%)
**Oral PrEP**				
Total N at follow-up	129	112	92	81
Persistent		109 (97%)	85 (92%)	70 (86%)
Switched or on another SC strategy		0	6 (7%)	10 (12%)
Discontinued SC strategy		3 (3%)	1 (1%)	1 (1%)
# still followed but missing data		9	7	6
# Loss of follow-up between this visit and the previous visit		8^a^	13	5
**Timed sex without condoms to peak fertility**				
Total N at follow-up	86	75	71	63
Persistent		63 (84%)	54 (76%)	48 (76%)
Switched or on another SC strategy		12 (16%)	14 (20%)	13 (21%)
Discontinued SC strategy		0	3 (4%)	2 (3%)
# still followed but missing data		9	3	5
# Loss of follow-up between this visit and the previous visit		2	1	3
**Wait to have a child**				
Total N at follow-up	27	27	25	24
Persistent		8 (30%)	3 (12%)	3 (12%)
Switched or on another SC strategy		18 (66%)	20 (80%)	17 (71%)
Discontinued SC strategy		1 (4%)	2 (8%)	4 (17%)
# still followed but missing data			1	
# Loss of follow-up between this visit and the previous visit			1	1

^a^2 of these were not LTFU but became pregnant

### Switch of Selected Safer Conception Strategies

Among women who did not continue a selected strategy, we describe what methods, if any, women reported using subsequent to discontinuation of the strategy at months 3,6 and 9 of follow-up (Table 3). The proportion of women who reported switching from oral PrEP to another safer conception strategy or using another strategy was 0% at 3 months, 7% at 6 months, and 12% at 9 months. The proportion of women who reported switching from timed sex without a condom to peak fertility to another safer conception strategy or using another strategy was 16% at 3 months, 20% at 6 months and 21% at 9 months. The proportion of women who reported switching from waiting to have a child to another safer conception strategy or using another strategy was 66% at 3 months, 80% at 6 months, and 71% at 9 months.

**Table 3:** Predictors of persistence on any form of safer conception strategy through 9 months (N = 163)

Participant Characteristics	N (%) or Median (IQR)		Univariate		Multivariable	
	Persistent	Non-persistent	Risk ratio (95% CI)	p-value	Risk ratio (95% CI)	p-value
Number of participants	121 (74.2%)	42 (25.8%)				
Age, years (n = 163)	24.8 (22.4, 27.5)	25.1 (21.4, 27.2)	1.00 (0.98,1.03)	0.79		
Education (n = 163)						
Grade 7 (Standard 5)—Grade 11 (Standard 9)	12 (9.9%)	7 (16.7%)	ref		ref	
Grade 12 (Standard 10)	67 (55.4%)	17 (40.5%)	1.26 (0.88, 1.81)	0.20	1.10 (0.77, 1.59)	0.60
Post-grade 12 (University, College, Vocational)	42 (34.7%)	18 (42.9%)	1.10 (0.76, 1.62)	0.60	0.92 (0.62, 1.37)	0.69
Currently employed (n = 163)	41 (33.9%)	7 (16.7%)	1.22 (1.04, 1.45)	0.02	1.30 (1.07, 1.59)	0.01
Income, monthly (n = 121)						
< $116	23 (26.4%)	13 (38.2%)	ref		ref	
$116—$232	29 (33.3%)	13 (38.2%)	1.08 (0.79, 1.48)	0.63	1.06 (0.78, 1.43)	0.70
> $232	35 (40.2%)	8 (23.5%)	1.27 (0.96, 1.69)	0.09	1.14 (0.87, 1.49)	0.34
Prior pregnancies (n = 139)						
0	52 (42.9%)	19 (45.2%)	ref			
1	49 (40.5%)	13 (30.9%)	1.08 (0.89, 1.30)	0.43		
2 +	20 (16.5%)	10 (23.8%)	0.91 (0.68, 1.21)	0.52		
Have had a child with main pregnancy partner (n = 162)	21 (17.4)	11 (26.8%)	0.86 (0.68, 1.07)	0.18	0.87 (0.65, 1.18)	0.38
Sexual partners, past 3 months (n = 162)						
0 or 1	109 (90.1%)	36 (87.8%)	ref			
2 +	12 (9.9%)	5 (12.2%)	0.94 (0.68, 1.29)	0.70		
Used condom at last sex (main pregnancy partner) (n = 162)	31 (25.6%)	16 (39.0%)	0.84 (0.67, 1.06)	0.14	1.03 (0.80, 1.30)	0.84
Sexual Relationship Power Scale ^a^ (n = 163)	2.6 (2.4, 2.8)	2.7 (2.4, 2.8)	0.89 (0.72,1.11)	0.33		
Dyadic Trust Score ^b^ (n = 163)	29.0 (25.0, 35.0)	30.0 (27.0, 33.0)	1.00 (0.98, 1.01)	0.66		
Social support ^c^ (n = 163)	4.0 (4.0, 4.0)	4.0 (3.9, 4.0)	0.91 (0.67, 1.22)	0.53		
Any alcohol consumption, past year (n = 161)	61 (50.8%)	21 (51.2%)	1.00 (0.83, 1.19)	0.97		
Depression ≥ 1.75 ^d^ (n = 160)	8 (6.8%)	2 (4.9%)	1.08 (0.78, 1.49)	0.64		
HIV Knowledge Score ^e^ (n = 163)	16.0 (14.0, 18.0)	14.0 (11.0, 17.0)	1.05 (1.01, 1.08)	0.003	1.04 (1.01, 1.08)	0.02
HIV Stigma Score ^f^ (n = 163)	2.0 (0, 5.0)	1.5 (0, 4.0)	1.01 (0.98, 1.03)	0.59		
Perceived HIV Risk ^g^ (n = 163)	20.0 (18.0, 22.0)	20.0 (18.5, 21.0)	1.00 (0.97, 1.03)	0.91		

Covariates were selected into the multivariable regression where p < 0.25 in univariate regression analyses

^a^Developed by Pulerwitz et al., Sexual Relationship Power score assesses the relationship power dynamics around relationship control and decision-making. Higher scores denoted a higher level of power in the relationship around relationship control and decision-making

^b^Dyadic Trust Scale was used to assess the level of trust a person has in their relationship. Higher scores denoted a higher level of trust in the relationship

^c^Duke-UNC Functional support scale was used to measure social support. Higher scores denoted higher levels of social support

^d^Hopkins Symptoms Checklist was used to assess depression. A cut off point of ≥ 1.75 was categorized as presence of depressive symptoms

^e^Adapted from Carey and Schroder, and Matthews et al. HIV knowledge score was used to assess a person’s knowledge about HIV spread, transmission, and prevention. Higher scores denoted higher levels of knowledge about HIV spread, transmission, and prevention

^f^Developed by Visser et al., HIV Stigma Scale was used to assess perceptions of HIV stigmatizing attitudes within the community. Higher scores denoted higher levels of HIV stigmatizing attitudes within the community

^g^Developed by Napper et al. Perceived HIV Risk scale was used to assess perceptions of their risk of acquiring HIV. Higher scores denoted higher levels of perception of risk of acquiring HIV

### Predictors of Persistence Through 9 Months

Table 3 depicts Poisson regression models predicting persistence in one safer conception strategy from three months through 9 months. The percentage of women who reported persisting in any form of safer conception strategy through 9 months was 74.2%. Women who were currently employed were significantly more likely to report persisting in any form of safer conception method (RR = 1.30, 95% CI: 1.07–1.59, p = 0.01). Also, HIV knowledge was positively associated with persisting in any form of safer conception method (RR = 1.04, 95% CI: 1.01–1.08, p = 0.02). Persistence in the use of any form of safer conception method did not differ by other factors including age, income, alcohol consumption, number of sexual partners, sexual relationship power, HIV stigma, or perceived HIV risk.

## Discussion

In this study of South African women planning for pregnancy with a partner with HIV or of unknown HIV serostatus, oral PrEP was the most frequently reported safer conception strategy used, followed by timed sex without condoms to peak fertility. Approximately 74% of women who reported initiating any form of safer conception strategy persisted through 9 months. Among those who were not persistent, switching between PrEP to another strategy during the follow-up period was low. In contrast, a large proportion of women reported switching from waiting to have a child to another safer conception strategy during the follow-up period**.** Considering that most of the women in this study did not know their partner’s HIV status, this finding underscores the importance of understanding women’s preferences and providing different HIV prevention options to women of reproductive age living in HIV-endemic regions to minimize their risk of acquiring HIV and transmitting it to their infants.

Prior safer conception studies among people with HIV reported similar or lower rates in the uptake of timed sex without condoms to peak fertility as a safer conception strategy [13, 15, 28], while reports of oral PrEP uptake have been variable among women planning for pregnancy [3, 13]. Other past studies [13–15, 29] confirm our findings that suggest limited uptake of sperm washing as a safer conception strategy. In the future, safer conception programs may need to reevaluate whether promoting sperm washing is necessary for those who do not struggle with infertility, considering costs and limited adoption of this approach in this population.

Importantly, we found that being employed predicted the persistent use of any form of safer conception strategy (waiting to have a child, timed sex without condoms to peak fertility, or oral PrEP). Women who are employed may be less worried about meeting necessities of life such as food and housing [30]. They may be positioned to focus on pursuing HIV risk reduction strategies. Women who are employed might be more empowered in their relationships and have a greater degree of power to make decisions for themselves that would reduce their susceptibility to acquiring HIV [30, 31]. Lastly, women who are employed are more likely to be able to overcome certain barriers that might affect persistent use of safer conception strategies (such as lack of transportation or distance to clinic). In our study, only 26% of women were employed. Programs may need to offer additional supports to women without employment in order to support successful use of safer conception strategies.

We also found that increasing levels of knowledge about HIV was associated with persistent use of any form of safer conception strategy. This finding suggests the need for healthcare providers to screen women exposed to HIV for fertility intentions and HIV goals and provide adequate and targeted safer conception counseling and education around HIV transmission, prevention, and complications. Past studies have suggested that while healthcare providers discuss maternal and child health during periconception and pregnancy periods, there is less discussion around HIV transmission and prevention [32]. Also, very few HIV-exposed women receive safer conception counseling. In this present study, quarterly safer conception counseling was provided to women and that may have accounted for the relatively high persistence in the selected safer conception strategy. Strategies to promote persistence may consider optimal frequency and settings needed to provide safer conception counseling to women exposed to HIV. Additionally, providing women during routine clinic visits with a range of safer conception options to choose from could enhance persistence and reduce the risk of HIV acquisition and transmission to their infants [33]. Therefore, women planning for pregnancy should be counseled about and, if desired, offered new efficacious modalities such as long-acting injectable cabotegravir [34, 35].

Despite women participating in quarterly safer counseling sessions, still few reported their partner disclosing their HIV status even though the majority were unaware of their partners’ status at baseline. Studies have suggested that men with HIV have reproductive needs, which could be leveraged to promote HIV prevention [36–38]. Therefore, interventions geared toward promoting the involvement of male partners in safer conception care could enhance a thorough knowledge about HIV, disclosure of HIV status, and consistent use of safer conception strategies among HIV-exposed women planning for pregnancy.

Strengths of this study include the use of a sample of women of reproductive age with fertility intentions in an HIV endemic setting, coupled with the quarterly assessment of six safer conception strategies, and the assessment of potential predictors using validated instruments. A limitation of this study was that the assessment of the use and persistence of safer conception strategies was based on self-report (collected using the safer conception card), and we did not characterize the frequency of use, and the actual use of the selected strategy may have been different. Additionally, we did not assess prevention-effective persistence –an assessment of persistence on safer conception strategies based on the ongoing need for HIV prevention, when the risk of HIV exposure is high. Lastly, our study was not designed to examine the mediation effects of women’s characteristics on the persistent use of safer conception strategies. For instance, while women’s HIV risk perception was low and dyadic trust was good, women still reported persistent use of safer conception strategies through 9 months of follow-up. Further investigation is therefore needed to better understand direct and indirect factors associated with persistent use of safer conception strategies.

In conclusion, oral PrEP and timed sex without condoms to peak fertility were the most frequently selected safer conception methods among HIV-exposed women planning for pregnancy. Further, HIV knowledge and employment predicted persistence on the same safer conception strategy. These findings suggest a need for interventions and policies facilitating HIV knowledge and employment opportunities for women of reproductive age living in an HIV endemic region. Clinic-based interventions should prioritize providing comprehensive education on the prevention and transmission of HIV and offer safer conception counseling and support prior conception and during pregnancy to HIV-exposed women and their male partners. Possible future studies could consider adapting and implementing innovative strategies to promote HIV awareness and access to various safer conception strategies for couples in South Africa planning for pregnancy.

## Data Availability

Not applicable
